# Structure-based peptide design targeting intrinsically disordered proteins: Novel histone H4 and H2A peptidic inhibitors

**DOI:** 10.1016/j.csbj.2021.01.026

**Published:** 2021-01-21

**Authors:** Kanin Wichapong, Carlos Silvestre-Roig, Quinte Braster, Ariane Schumski, Oliver Soehnlein, Gerry A.F. Nicolaes

**Affiliations:** a Department of Biochemistry, Cardiovascular Research Institute Maastricht (CARIM), Maastricht University, Maastricht, the Netherlands; b Institute for Cardiovascular Prevention (IPEK), LMU Munich Hospital, Munich, Germany; c German Center for Cardiovascular Research (DZHK), Partner Site Munich Heart Alliance (MHA), Munich, Germany; d Department of Physiology and Pharmacology (FyFa), Karolinska Institute, Stockholm, Sweden

**Keywords:** Disordered proteins, Peptides, Computer-aided molecular design (CAMD), Protein-protein interactions (PPIs), Histones, Neutrophil extracellular traps (NETs), aMD, accelerated molecular dynamics, ARDS, acute respiratory distress syndrome, BFE, binding free energy, BRCA-1, breast cancer type1 susceptibility protein, CCL5, chemokine ligand 5, CHIP, cyclical histone H2A interference peptide, DC, decomposition, H2A, histone H2A, H2B, histone H2B, H3, histone H3, H4, histone H4, HIPe, histone inhibitory peptide, HNP1, human neutrophil peptide 1, IDPs, intrinsically disordered proteins, IDPRs, intrinsically disordered protein regions, MD, molecular dynamics, MM/GBSA, molecular mechanics/generalised born surface area, NETs, neutrophil extracellular traps, p53, tumor protein 53, PDB, protein data bank, PPIs, protein-protein interactions, PTP1B, protein tyrosine phosphatase 1B, SMCs, smooth muscle cells

## Abstract

A growing body of research has demonstrated that targeting intrinsically disordered proteins (IDPs) and intrinsically disordered protein regions (IDPRs) is feasible and represents a new trending strategy in drug discovery. However, the number of inhibitors targeting IDPs/IDPRs is increasing slowly due to limitations of the methods that can be used to accelerate the discovery process. We have applied structure-based methods to successfully develop the first peptidic inhibitor (**HIPe** - **H**istone **I**nhibitory **Pe**ptide) that targets histone H4 that are released from NETs (Neutrophil Extracellular Traps). HIPe binds stably to the disordered N-terminal tail of histone H4, thereby preventing histone H4-induced cell death. Recently, by utilisation of the same state-of-the-art approaches, we have developed a novel peptidic inhibitor (**CHIP** - **C**yclical **H**istone H2A **I**nterference **P**eptide) that binds to NET-resident histone H2A, which results in a blockade of monocyte adhesion and consequently reduction in atheroprogression. Here, we present comprehensive details on the computational methods utilised to design and develop HIPe and CHIP. We have exploited protein–protein complexes as starting structures for rational peptide design and then applied binding free energy methods to predict and prioritise binding strength of the designed peptides with histone H4 and H2A. By doing this way, we have modelled only around 20 peptides and from these were able to select 4–5 peptides, from a total of more than a trillion candidate peptides, for functional characterisation in different experiments. The developed computational protocols are generic and can be widely used to design and develop novel inhibitors for other disordered proteins.

## Highlights

• Intrinsically disordered proteins/protein regions (IDPs/IDPRs) are emerging drug targets.
• Lack of fast methods hinders the discovery of inhibitors for IDPs/ IDPRs.
• Fast and inexpensive structure-based approaches have been developed.
• The developed methods were applied to succesfully design inhibitors targeting the disordered tail of histone H4 and H2A.
• The presented methods can be widely used to identify inhibitors for other IDPs/IDPRs.

## Graphical abstract

**Figure:**
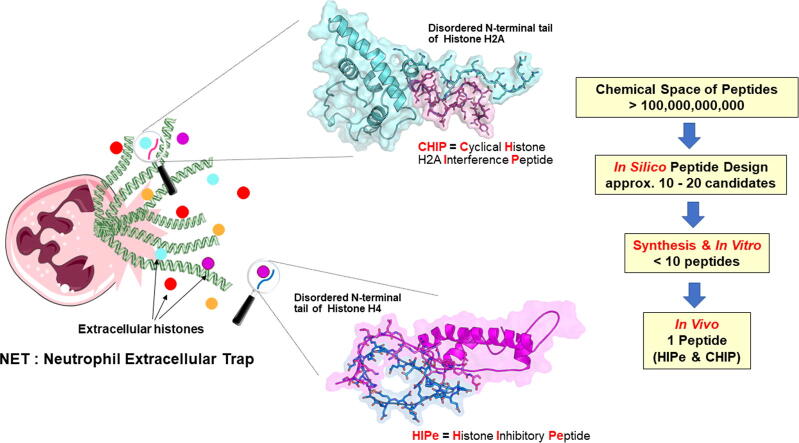

## 1 Introduction

Intrinsically disordered proteins (IDPs) and intrinsically disordered protein regions (IDPRs) are defined as proteins or segments of proteins that lack a stable three-dimensional (3D) folding structure [1], [2]. The discovery of IDPs and IDPRs has challenged and changed the paradigm in many different research fields such as molecular biology, protein structure study and especially in drug discovery [2], [3], [4], [5]. Earlier, it had been considered that a stable and fixed conformation of proteins determines their functions and roles in cell biology. However, an increasing number of studies have shown that IDPs/IDPRs also play crucial roles in various cell regulation mechanisms [1], [6], [7]. The functions of IDPs/IDPRs are diverse and can be classified as molecular recognition, molecular assembly, protein modification, and entropic chain activities [8], [9]. The disordered conformation is the key feature of IDPs/IDPRs that allows this class of proteins to bind with multiple protein partners with high specificity, and IDPs represent a central hub in protein–protein interactions (PPIs) networks [10], [11], [12]. Being a key regulator in cellular functions and a central hub in PPI networks, numerous IDPs and IDPRs are associated with and play a pivotal role in various human diseases e.g., cancer (p53, BRCA-1, PTP1B, c-Myc) [12], [13], [14], sepsis and inflammatory diseases (extracellular histones and NETs-resident histone) [15], [16], [17], [18], and neurodegenerative diseases (α-synuclein, Aβ, Tau) [19], [20]. Several inhibitors have been identified and developed against these proteins, and some compounds are now entering clinical trial phases [14], [21], [22]. Therefore, these studies and the successes of the development of inhibitors targeting disordered proteins are a strong evidence to show the proof of concept that targeting of IDPs/IDPRs is feasible and that they represent promising and emerging drug targets.

Due to the absence of stable secondary or tertiary structures and of well-defined binding pockets in IDPs/IDPRs, targeting of these types of proteins in drug discovery is a non-trivial and challenging task. To identify inhibitors for IDPs/IDPRs by using *in silico* methods, enhanced molecular dynamics (MD) simulations (e.g., replica exchange, accelerated MD or multiscale approaches) are employed to generate diverse conformations of disordered proteins [11], [23], [24], [25]. Subsequently, MD snapshots are analysed, and the conformations that contain transient druggable pockets will be exploited for structure-based virtual screening to identify potential candidates for IDPs/IDPRs. However, investigation of protein conformational landscapes can be time consuming and requires considerable computational resources. In addition, it is uncertain that the conformational space and landscape of the investigated proteins are completely explored despite the fact that long and rigorous simulation methods are employed. Thus, a fast approach such as by structure-based methods represents an alternative strategy that can be applied to develop inhibitors for targeting of disordered proteins. Though IDPs/IDPRs display distinct disordered structures, binding of IDPs/IDPRs with their binding partners (globular or other disorder proteins) can result in a stable conformation (disorder-to-order transition) [26], [27]. 3D structures of protein–protein complexes have been successfully utilised as a starting point to design and develop novel modulators (inhibitors or stabilisers) for several PPIs [28], [29], [30], [31], [32], [33]. Thus, we hypothesised that complexes between IDPs/IDPRs and their binding partners as well as the experimentally established interface interactions can be exploited as starting structures and guideline to design and develop inhibitors for IDPs/IDPRs.

To prove our concept, we have applied structure-based methods to develop novel inhibitors that target the disordered N-terminal tails of histone H4 and H2A. Our previous studies have revealed that electrostatic interactions between the highly positively charged N-terminal tail of histones H4, which are released from NETs (Neutrophil Extracellular Traps), with negatively charged phospholipids can induce cell death and promote chronic atherosclerosis [17]. Therefore, neutralisation of the N-terminal tail of histone H4 represents a promising approach to prevent cell lysis and tissues damage. To develop inhibitors to target the disordered N-terminal tail of histone H4, we have utilised the structural information on an existing protein–protein complex between histone H4 and its binding partner (histone acetyltransferase) as a starting structure to design and develop the first peptidic histone H4 inhibitor [17]. Recently, we have demonstrated that NET-resident histone H2A can interact with monocytes in a charge-dependent manner which consequently accelerates the progression of atherosclerosis upon acute infection. Again, we have applied structure-based approaches to design and identify peptides that can bind to the disordered tail of histone H2A thereby blocking the interactions with monocytes [18].

Here, we present comprehensive computational methods utilised to design and develop inhibitors to target the disordered region at the N-terminal tail of histone H4 and H2A. Similar computational methods that we have previously used to develop peptides to interrupt PPIs (CCL5-HNP1 complex) [28], [33] were also applied here to generate inhibitors for histone H4 and H2A. The protein–protein complexes of histone H4/H2A and their binding partners were exploited as starting structures to *in silico* rationally design peptidic inhibitors. Then, binding affinity between the designed peptides and histone H4/H2A were predicted by use of a molecular mechanics/generalised Born surface area (MM/GBSA) method, which is a fast and simple method to predict binding mode and binding affinity of protein–protein/peptide complexes [32], [33], [34], [35], [36]. The candidate peptides were prioritised and selected according to their predicted binding affinities for synthesis and further experimentally tested to investigate their inhibitory activities. By using this strategy, we have successfully developed the first peptidic inhibitor for histone H4 (**HIPe** - **H**istone **I**nhibitory **Pe**ptide) which exhibits therapeutic benefits in an atherosclerotic mouse model [17]. Likewise, the developed histone H2A inhibitor (**CHIP**- **C**yclical **H**istone H2A **I**nterference **P**eptide) is able to block monocyte adhesion in *in vitro* experiments and a related animal model [18]. The computational methods presented here are fast, straightforward and inexpensive approaches which can be widely applied to accelerate the development of novel inhibitors to interrupt PPIs as well as to target IDPs/IDPRs.

## 2 Results

### 2.1 Binding mechanism of histone H4 with membranes and description of the human histone H4-protein complex

**Fig. 1:**
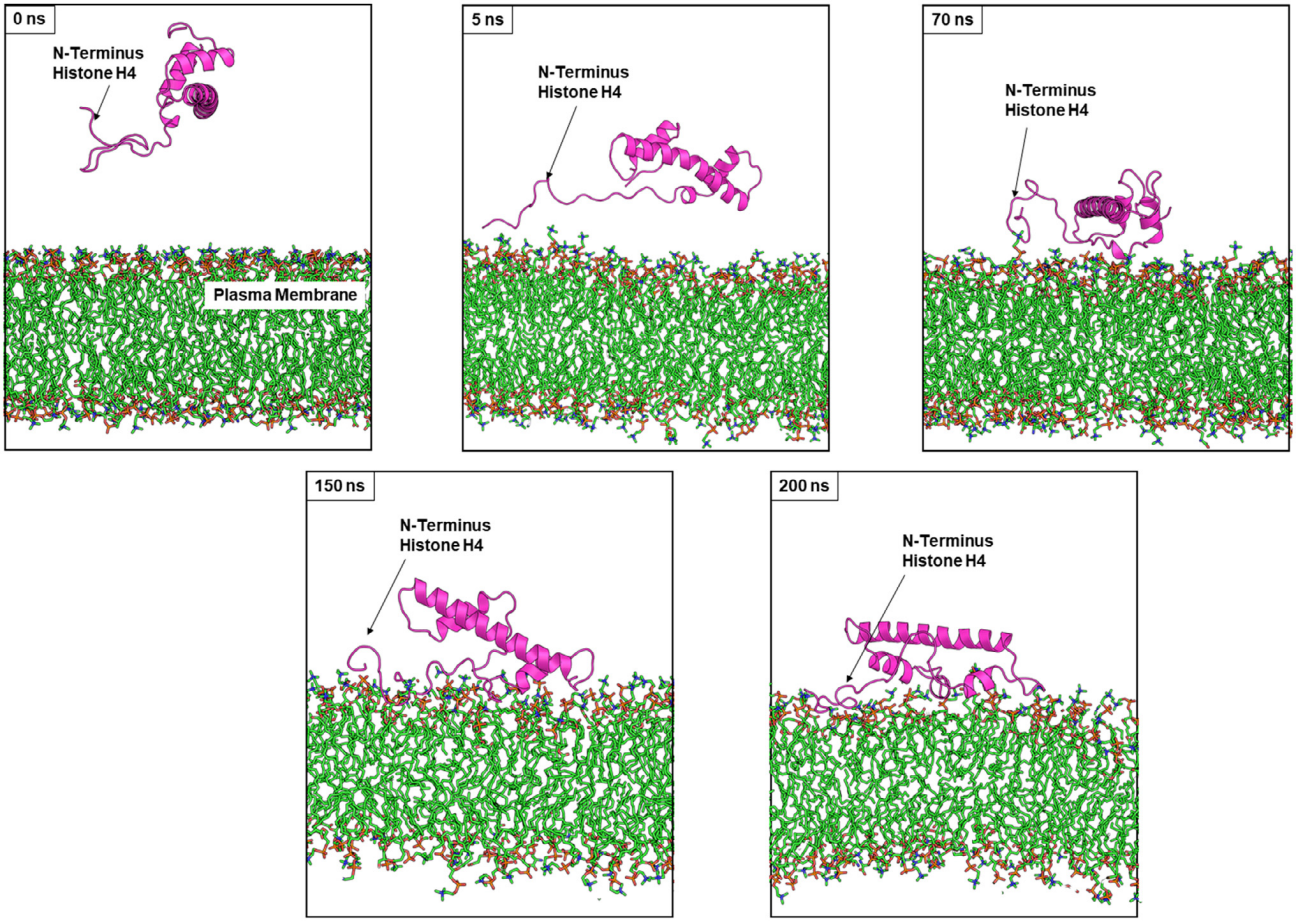
Binding mechanism of Histone H4 with plasma membranes extracted from different MD simulation time. Histone H4 is shown in magenta cartoon and membranes are displayed in green sticks. MD simulation revealed that the N-terminal tail of Histone H4 is major domain that binds and interacts with membranes. (For interpretation of the references to colour in this figure legend, the reader is referred to the web version of this article.)

Our previous study has revealed that histone H4 activates cell death by directly interacting with cell membranes which induces the formation of membrane pores [17]. To gain insight into the binding mechanism and to identify key residues of histone H4 that mediate the interaction of H4 with phospholipid membranes, MD simulations of the histone H4-membrane complex were performed. As displayed in Fig. 1, MD simulations revealed that histone H4 can bind membranes with different orientations but the N-terminal tail of histone H4 is consistently being the major part that binds and interacts with membranes. MD simulation results are in line with results derived from other experiments (e.g., confocal microscopy and bioinformatics approach) that indicate that the N-terminus (residue 1–24) is the main part of histone H4 that interacts with membranes. Moreover, different H4-derived peptides (i.e., residues 1–24 (the N-terminal domain), residues 25–68 (core domain), and residues 69–102 (C-terminal domain)) were tested in cytotoxicity assays to investigate their potential to induce cytotoxicity. Experimental results showed that the peptide derived from N-terminal tail by itself can induce cell death to the same extent as full-length histone H4. More details of these experiments and results can be found in our recent publication [17]. Thus, prevention of histone H4-membrane interactions by development of bioactive compounds to stably bind and neutralise the unstructured N-terminal tail of histone H4 represents a promising strategy to reduce histone H4-induced cytotoxicity.

**Fig. 2:**
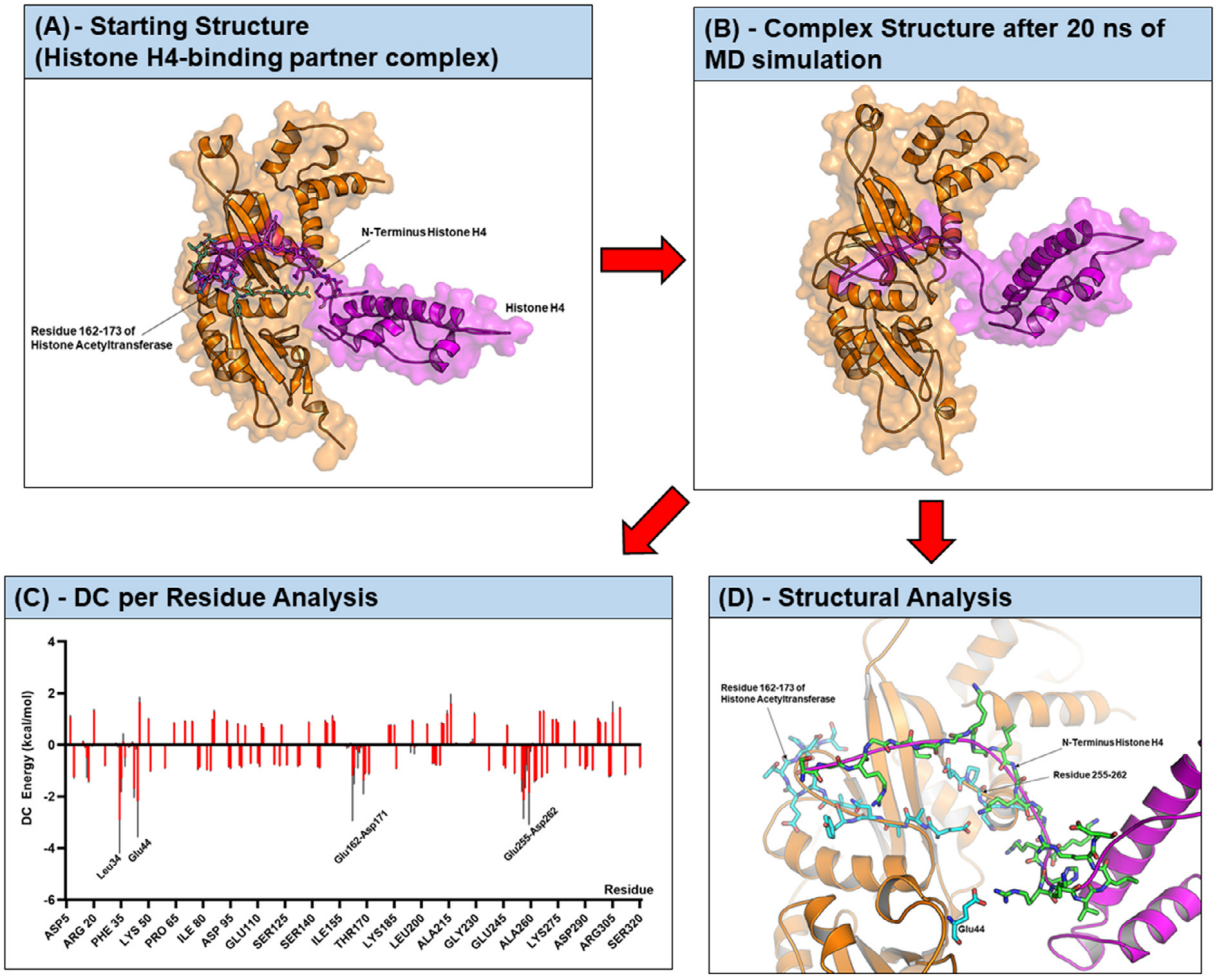
A structure-based method utilised for design peptidic inhibitors targeting histone H4. (A) Identification (or generation) of a likely binding mode between histone H4 and its binding partner (histone acetyltransferase) (B) MD simulation of the identified complex to investigate the stability of the complex and examine key interactions at the interface, (C) Decomposition (DC) energy per residue analysis together with (D) Structural analysis to identify key residues of the binding partner for interacting with the target protein (histone H4). The peptide fragment (e.g., residue 162 to 173, EAANYIDETDPS) from the binding partner was used as a starting peptide.

Next, we applied structure-based approaches to develop novel histone H4 inhibitors by first selecting a suitable complex between N-terminal histone H4 and one of its binding partners in the Protein Data Bank (PDB). The crystal structure of histone acetyltransferase in complex with *Ophiophagus hannah* histone H4 (PDB ID: 4PSW) was chosen because in this structure residues 9–24 of histone H4, covering around 66% of the N-terminal tail, binds into the binding pocket of histone acetyltransferase. Thus, this structure represents a suitable template to build a model of human histone H4 in complex with its binding partner which can be used for inhibitors design. In order to generate a complex between Homo sapiens histone H4 and histone acetyltransferase, we then docked 5 different conformations of Homo sapiens histone H4 (chain B of PDB ID: 1KX5) which were derived from MD simulations onto the binding pocket histone acetyltransferase. The docking results (around 70 poses) were visually inspected and the poses in which the N-terminal tail of histone H4 fitted into the binding pocket of histone acetyltransferase were selected. Based on this criterion, in total 33 different poses (binding modes are shown in supplement Figure SI-1) were chosen. Then, the derived docking complexes were optimised by performing a short MD simulation (500 ps) and binding free energy (BFE) of these complexes (supplement Table SI-1) were computed. The binding mode number 18 (as displayed Fig. 2 (A)) gave the lowest binding free energy (−79.84 ± 3.96 kcal/mol) indicating that this complex is the most energetically and thermodynamically favourable conformation of the ones tested. Therefore, complex number 18 represents a likely binding mode between human histone H4 and histone acetyltransferase. This complex was selected for further investigation to identify key residues and interactions between N-terminal histone H4 and its binding partner which were then utilised to guide the rational design of histone H4 inhibitors.

### 2.2 Design of peptide to neutralise N-terminal tail of histone H4

**Fig. 3:**
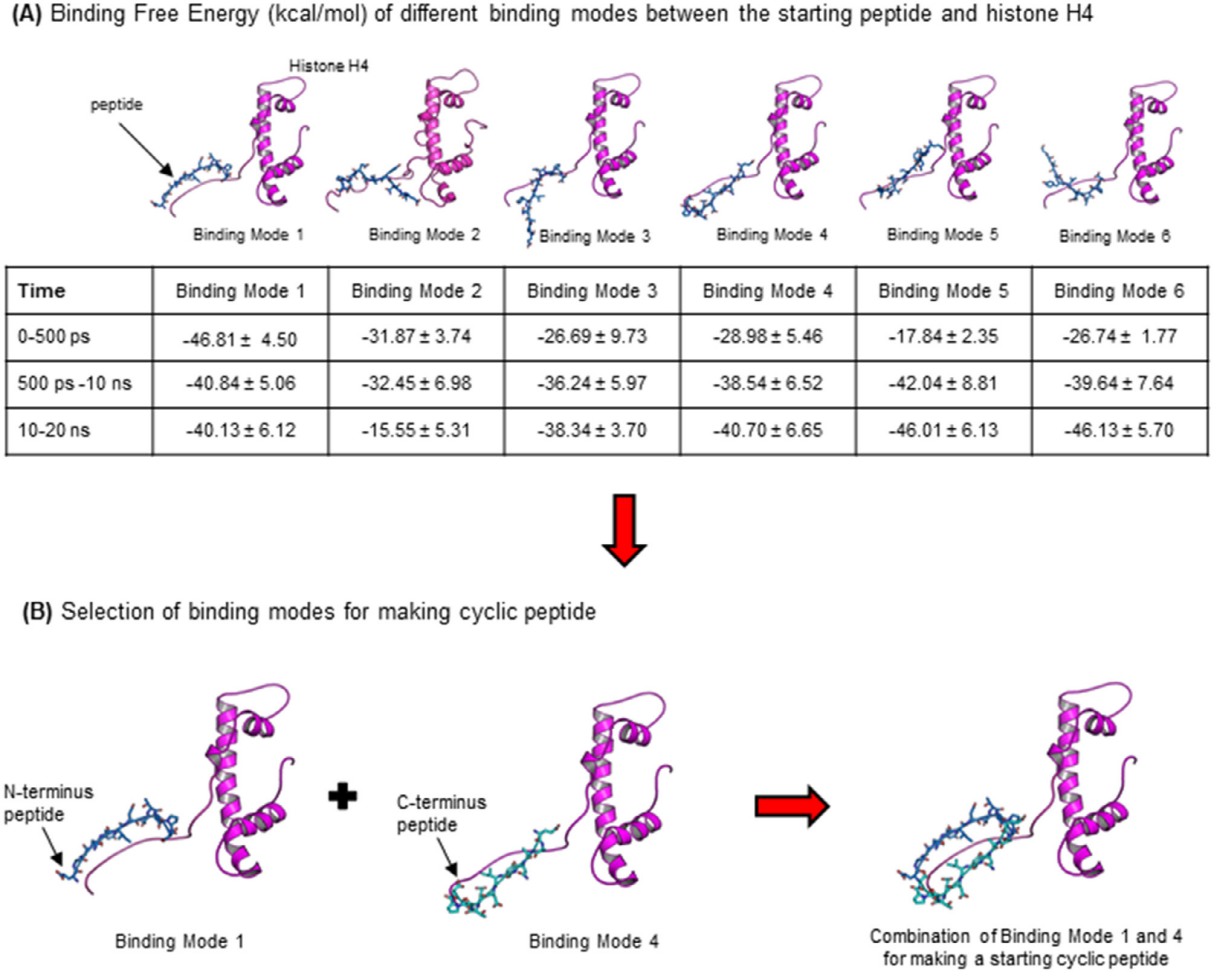
A schematic representation of approach applied to create a starting cyclic peptide. (A) The linear template peptide (EAANYIDETDPS) was docked on the N-terminal tail of Histone H4 and the derived binding poses were subjected for MD simulations (20 ns) and binding free energy calculation. Results are summarised in the table. (B) Binding mode 1 and binding mode 4 were selected and combined to build a cyclic peptide.

MD simulation of the selected complex (complex number 18) was extended for another 20 ns and the last MD snapshot is displayed in Fig. 2(B). By combination of results derived from decomposition (DC) energy per residue analysis (Fig. 2(C)) and structural analysis (Fig. 2(D)), we selected residues 162–173 (EAANYIDETDPS) from histone acetyltransferase as a starting peptide for further design and optimisation of peptidic inhibitors for histone H4. The binding mode of the starting linear peptide and N-terminal histone H4 was predicted by protein-peptide docking and 6 different binding modes (Fig. 3(A)) were obtained. Following our previously established protocols [33], a short MD simulation (500 ps) of these complexes was first employed. However, we observed that the complexes between a linear peptide and the unstructured tail of histone H4 were quite flexible during the simulations. Thus, we extended the simulations of these complexes till 20 ns, and snapshots derived from different MD periods (0–500 ps, 500 ps-10 ns, and 10–20 ns) were used for BFE calculation. Results as summarised in the Table in Fig. 3 (A) revealed that BFE of the binding modes 1, 3, 4, 5 and 6 derived from MD snapshots from 10 to 20 ns were comparable (approx. −40 kcal/mol). Interestingly, by investigation of these docking poses and their interactions with histone H4 we found that the binding modes 1 and 4 of this linear peptide bound to histone H4 in an opposite direction (Fig. 3(B)) while both binding poses yielded comparable BFE values. In the binding mode 1, the N-terminus of the peptide bound to the N-terminal tail of histone H4; while the C-terminus of the peptide in the binding mode 4 bound to the N-terminal tail of histone H4. We hypothesised that combination of these two linear binding modes, in the form of a cyclic, should improve binding affinity of peptides. Hence, we combined these two possible binding poses (binding mode 1 and 4 of the linear peptide) to construct a cyclic peptide (Fig. 3(B)).

The list and sequence of cyclic peptides are summarised in Table 1. Different strategies were applied to rationally *in silico* design cyclic peptides. For example, cyclic-1 was *in silico* designed by addition of cysteine residues at the N- and C-termini of each pose (binding mode 1 and 4) and then two linear peptides were linked together via disulfide bonds. Binding mode and interactions of cyclic-1 with histone H4 was investigated and specific residues at particular positions (e.g., I7E, T10E, T19E, and I22E in cyclic-2) were rationally mutated *in silico* in order to improve the BFE values with histone H4. It is known that peptides that contain two disulfide bonds (cyclic-1 and cyclic-2) may encounter difficulty during synthesis, such as in controlling the formation of correct disulfide bonds to prevent synthesis of mixed isomers. Thus, we generated new cyclic peptides (cyclic-3 to cyclic-9) by *in silico* mutation of cysteine residues at position 14 and 15 to serine, an amino acid with similar properties as cysteine, or to glycine/proline residue in order to constrain the cyclic conformation of peptides. Residues at position 14 and 15 were connected via peptide bond linkage, while the disulfide bond between cysteine residues at the N- and C-terminus was kept. Again, binding modes of these peptides with histone H4 were examined and specific residues were rationally mutated to increase the binding free energy with histone H4. For instance, investigation of binding poses of cyclic-5 with the N-terminal tail of histone H4 revealed that hydrophobic residues (e.g., proline or alanine residue) juxtapose positively charged residues (arginine or lysine residue) of histone H4. Thus, to improve interactions between peptides and histone H4, we *in silico* mutated proline and alanine residue to negatively charged residues (aspartic or glutamic residue) in cyclic-5 to cyclic-9. By doing this, we *in silico* designed in total 9 different cyclic peptides (Table 1).

**Table 1:** Sequence and binding free energy (kcal/mol) of designed peptides for histone H4.

Name	Sequence	S-S bond positions	Binding Free Energy (kcal/mol)
Cyclic-1	H-C^1^EAANYIDETDPSC^14^-OH H-C^15^SPDTEDIYNAAEC^28^-OH	C^1^-C^15^ C^14^-C^28^	−52.88 ± 7.06
Cyclic-2	H-C^1^EAANYEDEEDPSC^14^-OH H-C^15^SPDEEDEYNAAEC^28^-OH	C^1^-C^15^ C^14^-C^28^	−69.35 ± 8.31
Cyclic-3	H-C^1^EAANYIDETDPSSSEAANYIDETDPSC^28^-OH	C^1^-C^28^	−54.59 ± 7.67
Cyclic-4	H-C^1^EAANYIDETDPSGGEAANYIDETDPSC^28^-OH	C^1^-C^28^	−61.89 ± 7.30
Cyclic-5	H-C^1^EAANYEDEEDPSGGEAANYEDEEDPSC^28^-OH	C^1^-C^28^	−72.51 ± 9.21
Cyclic-6	H-C^1^EAANYEDEEDPSGGSPDEEDEYNAAEC^28^-OH	C^1^-C^28^	−75.76 ± 11.07
Cyclic-7	H-C^1^EAENYEDEEDDSPPEAENYEDEEDDSC^28^-OH	C^1^-C^28^	−85.22 ± 7.68
Cyclic-8	H-C^1^EAENEEDEDEDSPPEAENEEDEDEDSC^28^-OH	C^1^-C^28^	**−100.42 ± 8.50**
Cyclic-9	H-C^1^EAENYEDEEDDSPPSDDEEDEYNEAEC^28^-OH	C^1^-C^28^	−81.41 ± 9.72

These peptides were then docked onto the N-terminal tail of histone H4, which is extracted from the representative structure of human histone H4-histone acetyltransferase complex from the previous step (complex number 18), and we obtained in total 65 different poses of these 9 peptides. These 65 docking poses were subsequently subjected to MD simulations to optimise the docking poses and facilitate BFE calculation. To conduct MD simulations of these 65 poses (20 ns or longer per each pose) can be time consuming and we have recently demonstrated that trajectories derived from short MD simulations can be used for relative BFE calculation [33], [37], [38]. However, in such case of highly flexible protein-peptide complexes (i.e., the N-terminal tail of histone H4 and cyclic peptide), short MD simulations may lead to insufficient sampling of complex conformations for BFE calculation. It has been demonstrated however, that use of multiple short MD simulations from different protein–ligand (peptide) structures obtained by molecular docking can be applied to overcome this sampling issue [39], [40]. Thus, we applied these approaches to speed up the calculations. All of the derived docking poses of each peptide were subjected to MD simulations for 500 ps and binding free energies of each peptide (Table 1) were averaged over different binding poses of each peptide. Among these 9 peptides, 4 peptides which are cyclic-6 (BFE = −76 kcal/mol), cyclic-7 (BFE = −85 kcal/mol), cyclic-8 (BFE = −100 kcal/mol), and cyclic-9 (BFE = −81 kcal/mol) gave relatively low BFE values, implying that these peptides represent potential candidates for testing their biological activity to reduce the cytotoxicity of histone H4. By consideration of the BFE values and also the ease and feasibility of synthesis; therefore, these peptides (cyclic-6, 7, 8 and 9) were selected for synthesis and further functional characterisation.

### 2.3 Biological activity of designed peptides to prevent histone H4-mediated cell lysis

**Fig. 4:**
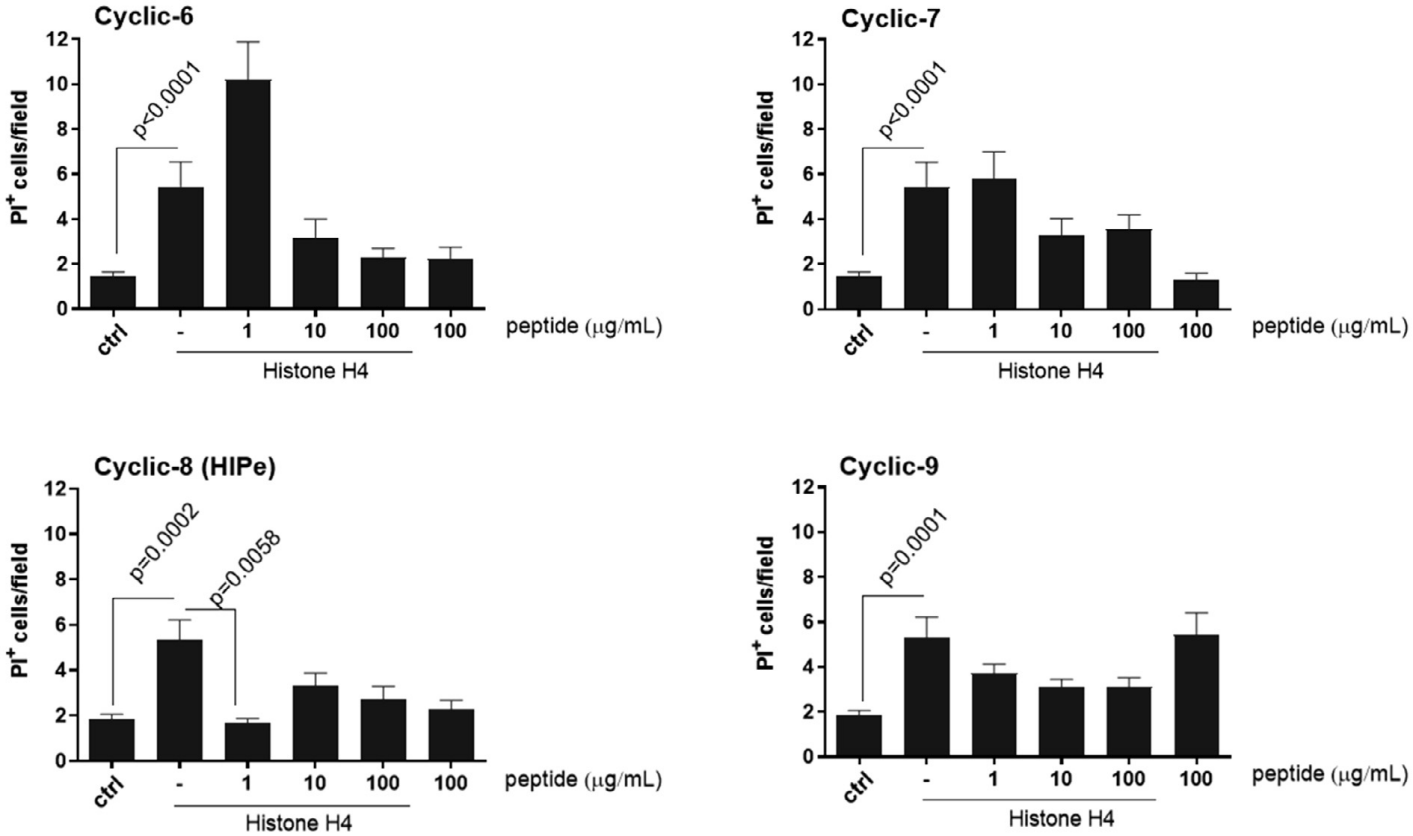
Cyclic peptides prevent histone H4-induced cell death. PI incorporation in SMCs treated with histone H4 (50 µg/mL) in the presence or absence with different peptides (cyclic-6, cyclic-7, cyclic-8, and cyclic-9). Data are mean +/− SEM. Kruskal–Wallis test with Dunn’s correction (>2 variables) was used and P values are reported, n = 19–24 fields per bar.

The biological activities of cyclic-6, −7, −8 and −9 for reducing histone H4-induced cytotoxicity are compared in Fig. 4. At the low peptide concentration (1 µg/mL), cyclic-8 exhibited the highest potential to prevent cell death. In fact, at such low concentration (1 µg/mL or 312.41 nM of cyclic-8), this peptide was able to fully reverse the cytotoxic ability of histone H4. On the other hand, cyclic-6 cyclic-7, and cyclic-9 did not exhibit inhibitory effects on histone H4-mediated cell death at such low concentration. Taken these results together, cyclic-8 is the most potent inhibitor to prevent histone H4-mediated cell death.

### 2.4 Prevention of histone H4 membrane binding by neutralisation of its disordered N-terminal tail by the designed peptide

**Fig. 5:**
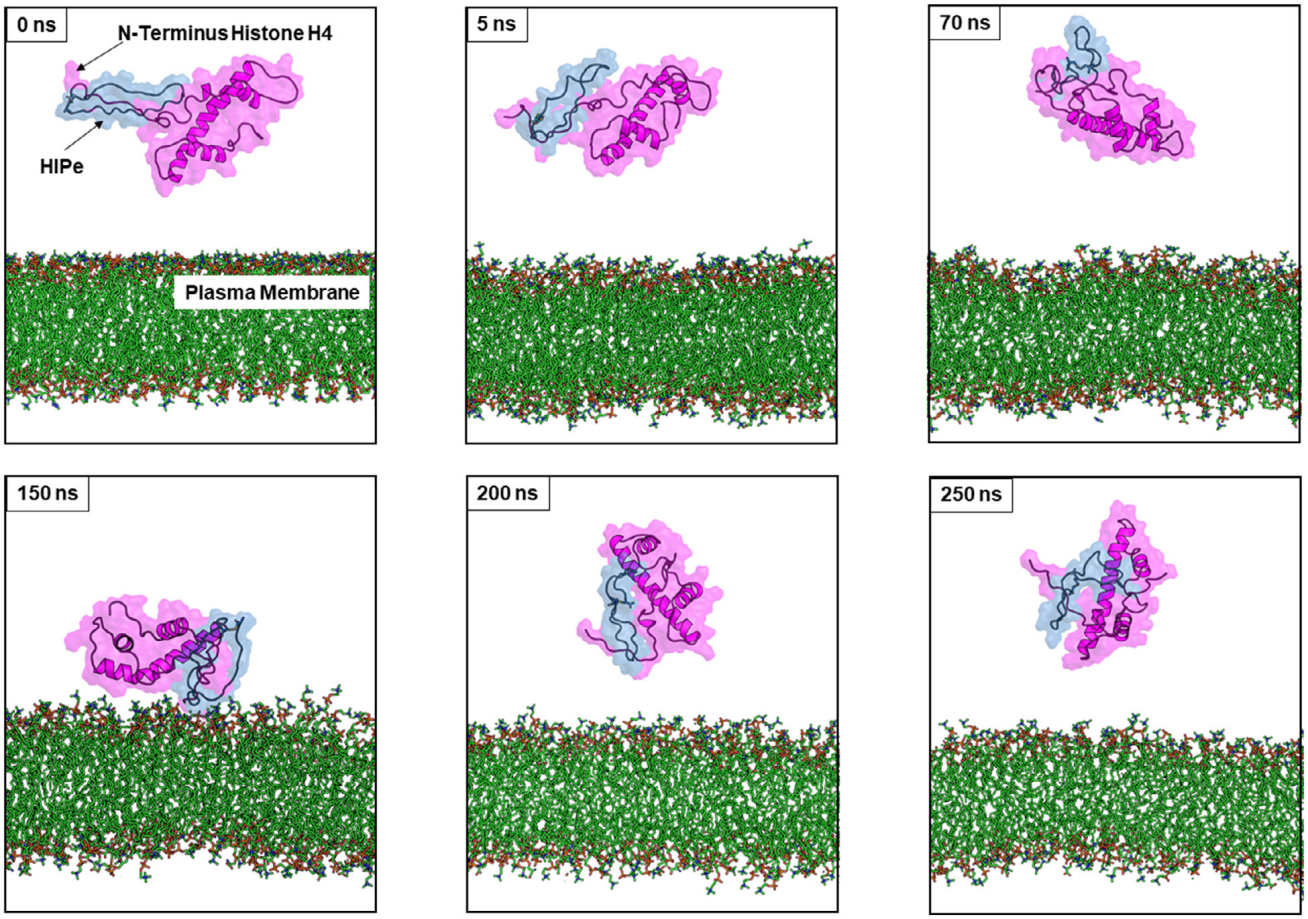
MD simulations of histone H4 in complex with HIPe (Histone Inhibitory Peptide) and membrane. HIPe can form a stable complex with histone H4 and consequently can prevent the interactions and binding between the histone H4 and membranes.

The specificity of the cyclic peptides (cyclic-6, −7, −8, and −9) with histone H4 was examined by calculation of the BFE of these peptides with different core histone (H2A, H2B, H3, and H4). Cyclic-8 not only showed high potential in prevention of histone H4-mediated cell death (*in vitro* cytotoxicity assay) but this peptide also exhibited specific binding with histone H4 as predicted by the relative *in silico* BFE, as indicating by the lowest BFE values of cyclic-8 with histone H4 than with other histone isotypes (H2A, H2B, and H3) (Table 2). Thus, cyclic-8 (**HIPe** - **H**istone **I**nhibitory **Pe**ptide) was selected for further *in vitro* and *in vivo* experiments. The experimental protocols and results of other related *in vitro* experiments have recently been published elsewhere and more details can be found there [17]. Briefly, we demonstrated that HIPe prevented the interactions between histone H4 and membranes as shown by confocal microscopy and consequently disrupted the histone H4-induced membrane pore formations as analysed by atomic force microscopy and live scanning ion conductance microscopy [17]. Additionally, MD simulations of histone H4 in complex with HIPe (Fig. 5) revealed that HIPe bound stably with the N-terminal tail of histone H4. The complex between histone H4 and HIPe touched (partially bound) the surface of the membrane at some time points during MD simulation (e.g., at 150 ns as displayed in Fig. 5); however, after extending the MD simulation to 200 ns the H4-HIPe complex moved away from the membrane surface (Fig. 5). The MD simulation of the histone H4-HIPe complex was prolonged to 250 ns and we still observed that the histone H4-HIPe complex continued to move away from the membrane surface. This can be due to the negatively charged residues (glutamic (E) and aspartic (D) acid) of HIPe (H-C^1^EAENEEDEDEDSPPEAENEEDEDEDSC^28^-OH) that exhibit strong repulsive interactions with phospholipids which consequently prevent the histone H4-HIPe complex from binding to membranes. These negatively charged residues of HIPe also contribute to strong interactions and binding with the N-terminal tail of histone H4 (^1^SGRGKGGKGLGKGGAKRHRKVLRD^24^), containing several positively charged residues (4 arginine (R) and 5 lysine (K) residues), which result in the establishment of a stable complex between histone H4 and HIPe. Finally, we investigated the potential of HIPe to abrogate histone H4-induced SMC cell death and experimental results confirmed that treatment by HIPe significantly reduced the amount of cell death to a similar extent as control experiments in which antibodies were used to ligate the histone H4 N-terminus. Furthermore, administration of HIPe showed therapeutic benefits in a histone-dependent mouse model of atherosclerosis [17]. To prove the specificity of the sequence of HIPe for binding with histone H4, we also experimentally tested biological activity of a scrambled version of HIPe (sHIPe: H-CDDDPSANEEEEDEEEEDPNASDEEEEC-OH). Administration of sHIPe did not exhibit significant therapeutic effects [17], implying that histone H4 binds specifically to HIPe, Also, these results point out that although electrostatic interactions are the predominant driving force to assist the formation of a stable H4-peptide complex as discussed above, its amino acid sequence also plays an essential role for binding with histone H4. The sHIPe contains the same overall net charge but a different amino acid sequence as compared to HIPe, yet sHIPe cannot bind and neutralise histone H4 [17]. Taken together, we can conclude that the cyclic peptide HIPe is a potent histone H4 inhibitor that can be applied to prevent histone H4-induced cell death as was proven by the treatments with this peptide in various experiments (from *in vitro* to animal model).

**Table 2:** Predicted binding free energy (kcal/mol) of cyclic-6, −7, −8, and −9 with different histone subtypes.

	H2A	H2B	H3	H4
Cyclic-6	−75.63 ± 4.63	−54.94 ± 7.58	−49.15 ± 15.09	−75.76 ± 11.07
Cyclic-7	−72.96 ± 6.41	−63.64 ± 2.09	−62.96 ± 9.24	−85.22 ± 7.68
Cyclic-8	−95.34 ± 8.67	−91.62 ± 9.94	−63.49 ± 3.75	−100.42 ± 8.50
Cyclic-9	−79.93 ± 6.52	−60.63 ± 11.78	−55.09 ± 12.23	−81.41 ± 9.72

### 2.5 Design of peptides targeting histone H2A to interrupt monocyte adhesion

As shown in Table 2 above, we compared BFE values of cyclic-6 with histone H2A, H2B, H3 and H4, and we found that cyclic-6 gave the lowest BFE (with a small margin of standard deviation value) to histone H2A (−75.63 ± 4.63 kcal/mol) implying a favourable binding of this peptide to histone H2A. On the other hand, cyclic-7, −8, and −9 showed a preferred binding to histone H4 (lower BFE to histone H4 than other histone subtypes). To develop specific peptidic inhibitors for histone H2A, we therefore utilised the cyclic-6 as a starting peptide for further optimisation such as to provide a series of peptides with improved binding affinity for histone H2A. Four new peptides (cyclic 10–13) were generated based on cyclic-6. Their sequence and BFE value with histone H2A are summarised in Table 3. However, BFE values of these peptides (cyclic 10–13) are around −68 to −78 kcal/mol which are quite comparable to the template peptide (cyclic-6, BFE = −76 kcal/mol). Thus, we selected cyclic-6 for testing its activity in further experiments. Moreover, the availability of cyclic-7, −8, and −9 from the previous experiments also allowed us to test and compare the activity of these peptides in the *in vitro* adhesion assay.

**Table 3:** Sequence and binding free energy (kcal/mol) of designed peptides for histone H2A.

Name	Sequence	S-S bond positions	Average Binding Free Energy (kcal/mol)	Lowest Binding Free Energy (kcal/mol)
**Cyclic-6 as a template:** H-C^1^EAANYEDEEDPSGGSPDEEDEYNAAEC^28^-OH				
**Cyclic-10**	H-C^1^EAANEEDEEDPSGGSPDEEDEENAAEC^28^-OH	C^1^-C^28^	−78.05 ± 12.50	−91.36 ± 9.97
**Cyclic-11**	H-C^1^EAANEEDEEDPSGGSPDAEEEENAAEC^28^-OH	C^1^-C^28^	−75.12 ± 10.07	−89.09 ± 4.62
**Cyclic-12**	H-C^1^EAANYSDEEDPSGGSPEAESEYNAAEC^28^-OH	C^1^-C^28^	−70.42 ± 9.78	−82.77 ± 8.14
**Cyclic-13**	H-C^1^EAANYEDEEDPSGGSPAEADEYAAAEC^28^-OH	C^1^-C^28^	−68.02 ± 8.78	−84.71 ± 6.29
**PDB ID: 4QYL as a template**: - Residue 31–43 (^31^PVPLSEVPDYLDH^43^) and 78–91 (^78^NCLKYNAKDTIFYR^91^) of Bromodomain				
**Cyclic-14**	H-C^1^VPLSEVPDYLDHCCLKYNAKDTIFYC^26^-OH	C^1^-C^26^	−30.53 ± 8.76	−42.21 ± 3.51
**Cyclic-15**	H-C^1^VPLSEVPDYLDCCLKYNAKDTIFYC^26^-OH	C^1^-C^26^	−33.48 ± 14.44	−61.45 ± 3.98
**Cyclic-16**	H-C^1^VPLSEVPDYLDAALKYNAKDTIFYC^26^-OH	C^1^-C^26^	−24.12 ± 6.13	−30.63 ± 3.35
**Cyclic-17**	H-C^1^EPLSEVEDYLDSSLKYNAKDTINYC^26^-OH	C^1^-C^26^	**−48.74 ± 22.45**	**−79.63 ± 2.79**
**Cyclic-18**	H-C^1^EALSEVPDYEDPPLADNAADTIFYC^26^-OH	C^1^-C^26^	−37.74 ± 12.58	−60.80 ± 10.09
**Cyclic-19**	H-C^1^EALSEVADYEDPPLADNAADTIYYC^26^-OH	C^1^-C^26^	−40.67 ± 11.53	−60.97 ± 3.75
**Cyclic-20**	H-C^1^EPLEAEEDYLDSSEAYNDKDTINYC^26^-OH	C^1^-C^26^	−40.17 ± 16.76	−58.18 ± 6.75
**Cyclic-21**	H-C^1^EPLEAAEDYLDSSEAENEKDTENYC^26^-OH	C^1^-C^26^	−37.83 ± 10.39	−56.28 ± 5.87

**Fig. 6:**
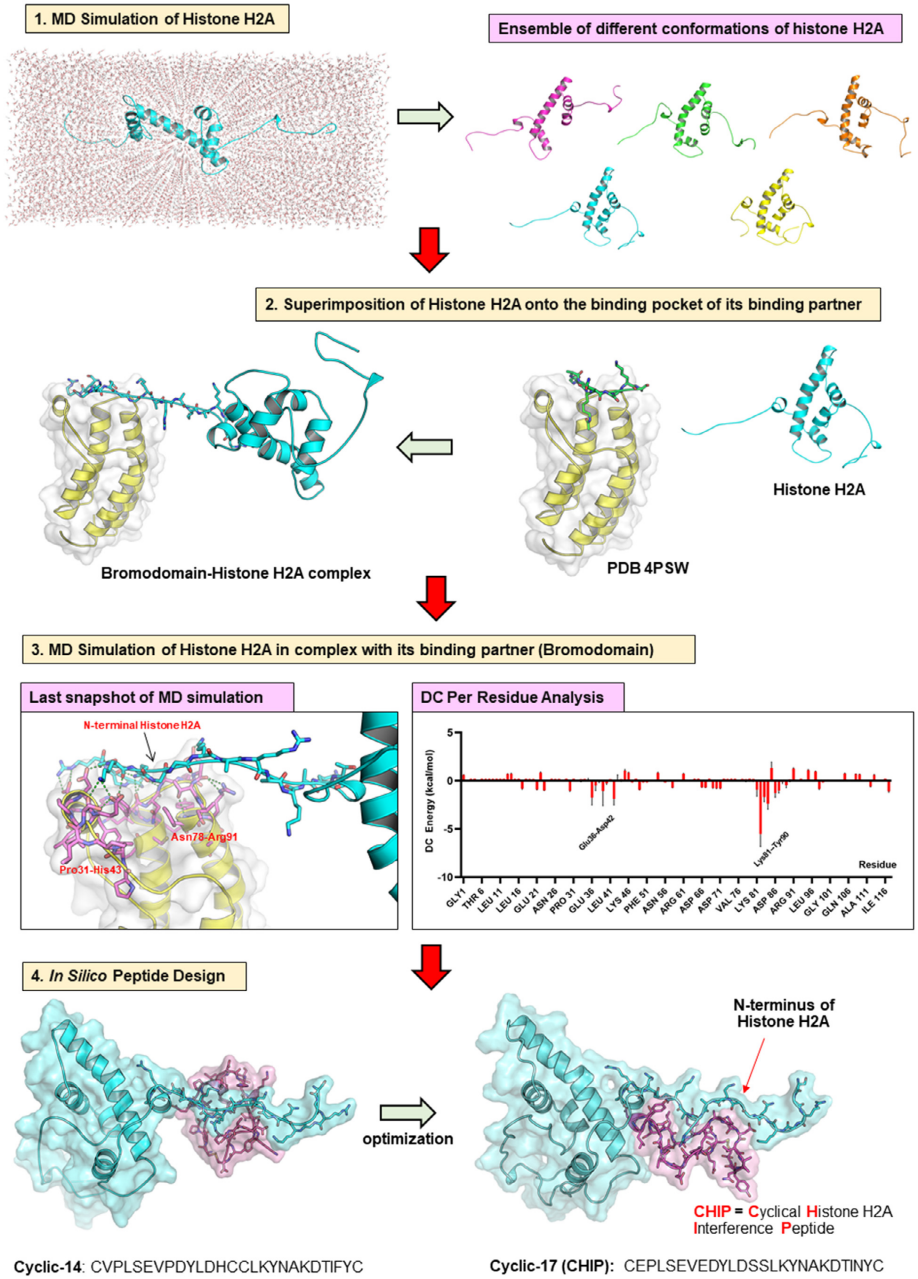
A workflow of computer-based methods used to develop histone H2A peptidic inhibitors. (Step 1), MD simulation of free histone H2A was performed to generate ensemble of different of conformations of histone 2A. These conformations were visually inspected. (Step 2), structures of histone H2A showing linear conformation at the N-terminal tail were selected and then were superimposed onto the histone H2A-derived peptide in complex with bromodomain (PDB ID: 4QYL). (Step 3), A selected complex of histone H2A and bromodomain derived from the previous step was subjected to MD simulations (20 ns). Decomposition (DC) per residue analysis and structural analysis were employed to identify key residues for interacting with histone H2A. (Step 4), Cyclic-14, which was derived from residue 31–43 (^31^PVPLSEVPDYLDH^43^) and 78–91 (^78^NCLKYNAKDTIFYR^91^) of bromodomain, was used as a starting structure for further peptide design and optimisation. The most potent peptide (Cyclic-17 or CHIP) binds to the N-terminal tail of histone H2A.

Since *in silico* optimisation of cyclic-6 did not result in an improvement of BFE of peptides (cyclic 10–13) with histone H2A, identification of a new template peptide having a sequence different from HIPe represents an alternative approach to develop potent inhibitors for histone H2A. Therefore, as an alternative strategy, we searched for the experimental structure of a suitable complex between histone H2A and its binding partners from the PDB. We selected and utilised a histone H2A-derived peptide-bromodomain complex (PDB ID: 4QYL) to rationally design and develop peptidic inhibitors of histone H2A by using similar structure-based approaches. A schematic representation of the structure-based workflow to generate histone H2A inhibitors is displayed in Fig. 6. First, MD simulations of human histone H2A (chain C from the nucleosome structure, PDB ID: 1KX5) were performed for 100 ns. Then, MD snapshots were inspected and the structures that showed linear conformations of the N-terminal tail of histone H2A, which can fit into the binding pocket of the template complex, were selected (step 1, Fig. 6). Since the selected co-crystallised structure (PDB ID: 4QYL) is the complex between linear peptide derived from human histone H2A and the human bromodomain, we thus superimposed the structure of the selected MD snapshots of histone H2A onto the experimental X-ray structure (step 2, Fig. 6). The complex that shows an optimal fit between the N-terminal tail of histone H2A onto the binding surface of the bromodomain, as determined by the low root-mean-square deviation (RMSD) values compared to the template (4QYL), was selected and was subsequently subjected to MD simulation (20 ns). Again, DC per residue analysis together with structural analysis were employed to investigate and identify key residues of the bromodomain that interact with the N-terminal tail of histone H2A (step 3, Fig. 6). Structure analysis results reveal that the loop regions (residues 31–43 (^31^PVPLSEVPDYLDH^43^) and 78–91 (^78^NCLKYNAKDTIFYR^91^)) of bromodomain are the major parts that contribute to the interactions with the N-terminal tail of histone H2A. Thus, we utilised these loops as a template for the design of peptidic inhibitors of histone H2A. Eight peptides were generated by use of a rational approach as previously explained in section 2.2 and then docked onto the N-terminal tail of histone H2A. BFE value averaging from different binding poses of each peptide (as previously done for calculating BFE of peptides with histone H4) are summarised in Table 3. Among these eight peptides (cyclic 14–21), cyclic-17 gave the lowest BFE value (−49 kcal/mol). The average BFE values of the cyclic 14–21 are relatively high (around −25 to −49 kcal/mol) compared to cyclic 10–13 (approx. −68 to −78 kcal/mol). We therefore considered BFE value of each individual docking pose for each peptide and found out that there is one docking pose of cyclic 17 that exhibited a strong binding with histone H2A (BFE = -79 kcal/mol). From these derived results, we therefore selected cyclic-17 to investigate its potential to neutralise histone H2A and prevent monocyte adhesion.

### 2.6 Prevention of monocyte adhesion by peptidic histone H2A inhibitors

**Fig. 7:**
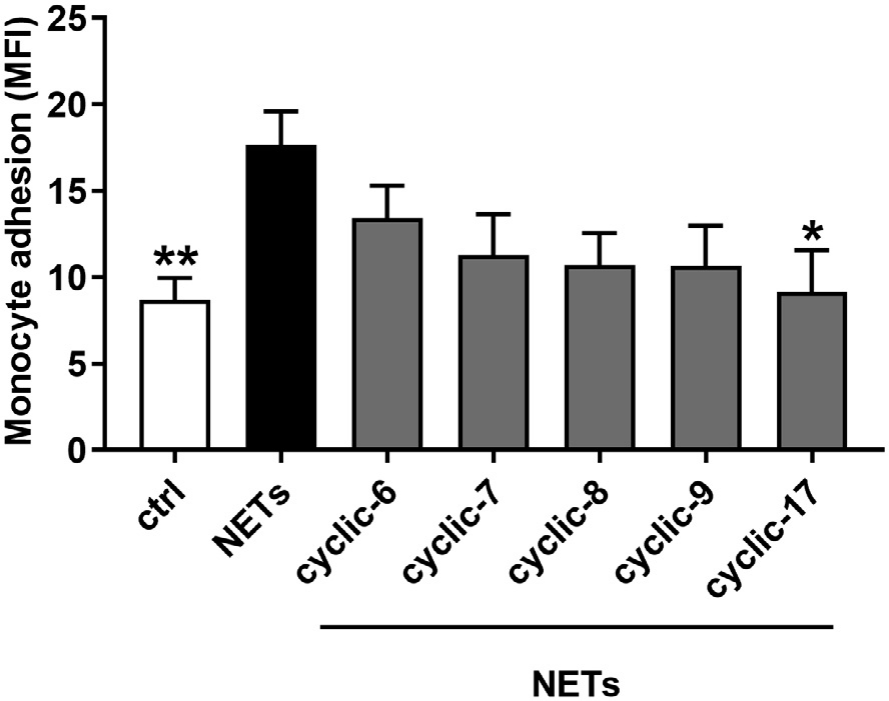
Cyclic peptides targeting NET-resident H2A disrupt monocyte adhesion that is promoted by NETs. *In vitro* static adhesion assay to monitor monocyte adhesion to NETs. Human classical monocytes were added to immobilized neutrophils (ctrl) or to NETting neutrophils (induced by A23187) and monocyte adhesion was quantified in a fluorescence plate reader as mean fluorescence intensity (MFI). To study monocyte adhesion to NETs and to investigate the ability of cyclic-6, −7, −8, −9 and −17 (CHIP) to block this process, NETs were preincubated with these peptides at the concentration of 200 µg/ml. Data are mean +/- SEM. One-way Anova. **p < 0.01, *<0.05 vs. NETs. n = 5–14 per bar.

We selected five peptides (cyclic-6, −7, −8, −9 and −17) to investigate their ability to disrupt monocyte adhesion to NETs (Fig. 7). Induction of NETs promoted an increase in monocyte adhesion (Fig. 7), an increase we previously reported to be reverted upon DNase digestion of NETs or antibodies targeting NET-resident histone H2A [18]. To test the impact of the five candidate peptides, NETs were preincubated with the peptides prior to addition of monocytes. As shown in Fig. 7, all five cyclic peptides can reduce monocyte adhesion, and among these five candidates, the cyclic-17 was most potent. Thus, the cyclic-17, which is also called **CHIP** (**C**yclical **H**istone H2A **I**nterference **P**eptide), represents our most potent peptic inhibitor to prevent monocyte adhesion by targeting NET-resident H2A. We further tested the inhibitory activity of CHIP in different experimental settings including by atomic force microscopy and in *in vivo* models of atherosclerosis, the results of which are detailed in our recent publication [18]. Briefly, CHIP can diminish the strength of the interaction between monocytes and NETS, and neutralization of NET-resident H2A by CHIP shows therapeutic benefits by reducing arterial adhesion as well as decreasing atherosclerotic lesion sizes in a model of LPS-accelerated atherosclerosis [18].

## 3 Discussion

The targeting IDPs/IDPRs is an emerging field in drug discovery and development. However, the number of available IDPs/IDPRs inhibitors is increasing only slowly due to a lack of adequate computational methods to accelerate the identification of inhibitors for IDPs/IDPRs. Recently, an ensemble-based thermodynamic framework was developed and successfully applied to analyse the apparent binding affinity between IDP (c-Myc) and ligands [41]. Here, we present an alternative approach by application of structure-based methods that use the structural information of protein–protein complexes to develop novel modulators (inhibitors or stabilisers) for PPIs [32]. Previously, we have used this approach to successfully develop peptidic inhibitors to interrupt the interactions between CCL5 and HNP1 thereby preventing their complex formation [28], [33]. In this work, we extend the applications of the structure-based method to challenging targets which are the disordered regions at the N-terminal tail of histone H4 and H2A.

**Fig. 8:**
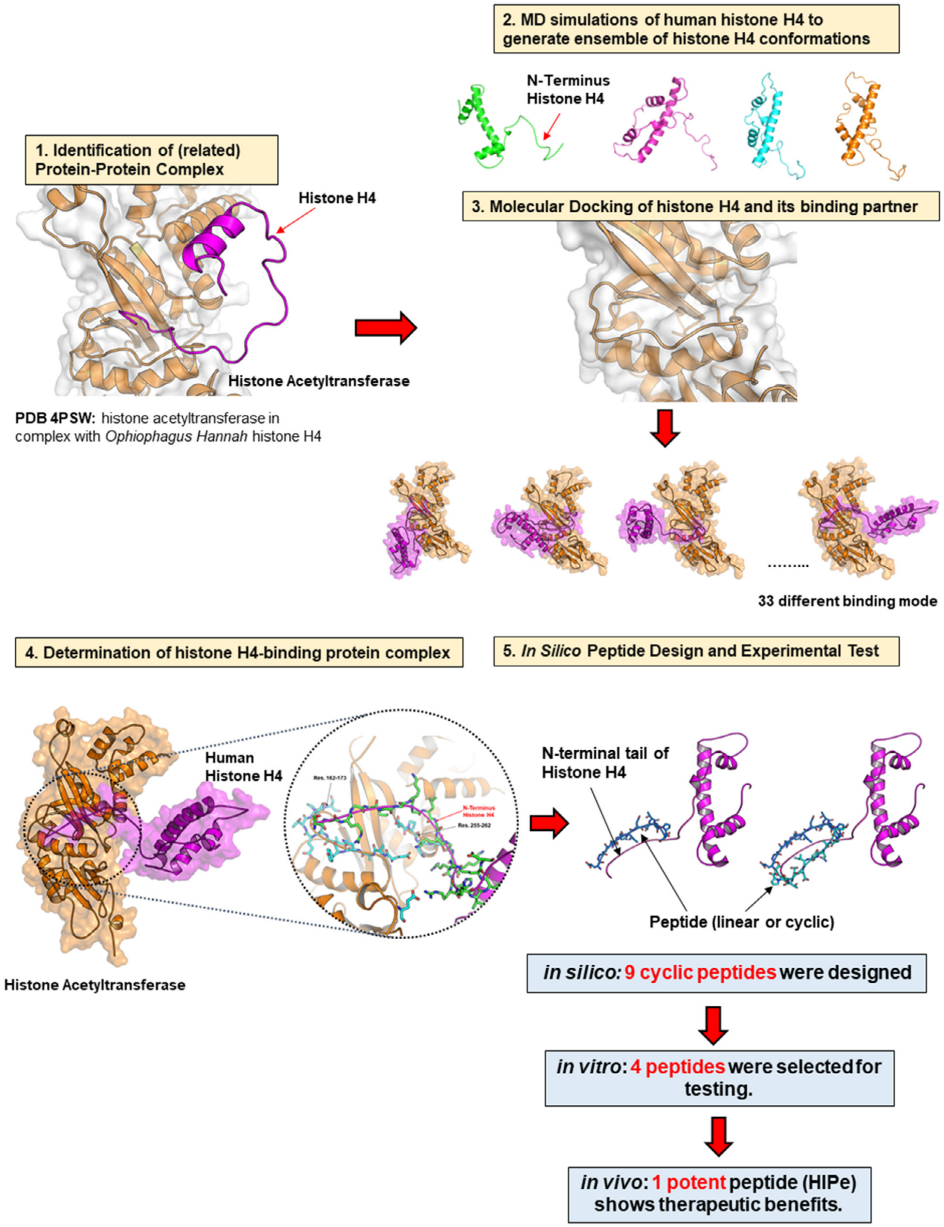
A schematic representation of the workflow utilised to design and develop histone H4 peptidic inhibitors. (Step 1), The complex between the related histone H4 and its binding partner was searched and selected from the PDB. The crystal structure of histone acetyltransferase in complex with *Ophiophagus Hannah* histone H4 (PDB ID: 4PSW) was chosen in this work. (Step 2), MD simulation of free histone H4 was performed to generate different conformations of human histone H4. (Step 3), The structures of histone H4 derived from step 2 were docked on the binding pocket of the histone acetyltransferase. (Step 4), A likely binding mode of histone H4 and histone acetyltransferase was determined based on binding free energy. (Step 5), A complex obtained from step 4 was investigated for key interacting residues and then exploited as a starting structure for peptide design. 9 different cyclic peptides were *in silico* designed and their binding strength with histone H4 was predicted by binding free energy calculation. 4 cyclic peptides with low binding free energy were selected for *in vitro* assay testing and finally, the most potent inhibitor (HIPe) was tested for its potential to disrupt the interactions between histone H4 and membrane and also its therapeutic benefits in atherosclerotic mouse model.

A schematic representation of the workflow used to design and develop histone H4 inhibitors is demonstrated and explained in Fig. 8. We have applied a combination of structure-based approaches and fast computational techniques (i.e., molecular docking, short MD simulations, and inexpensive BFE methods) to rational *in silico* design of various candidate peptides. As in our previous work [33], [37], [38], we focus exclusively on the relative BFE to predict and prioritise the binding strength of newly designed inhibitors of the target proteins. Hence, an inexpensive and fast method that combines a short MD simulation to optimise structures and subsequent MM/GBSA was applied for this purpose. We selected 4 peptides which gave a relatively low BFE (more negative values) for further synthesis and *in vitro* experiment (cell viability assay). The most potent peptide (HIPe) can prevent histone H4-induced cell lysis by stably binding with the N-terminal tail of histone H4 and consequently blocking the interactions between histone H4 and membranes. Moreover, the HIPe shows therapeutic benefits in a mouse model of atherosclerosis [17]. Although the sequence of HIPe (H-CEAENEEDEDEDSPPEAENEEDEDEDSC-OH) is very different from the original starting sequence (residues 162–173: EAANYIDETDPS), the structure-based method presented in this work is extremely useful since it offers a rational starting point for peptide design and helps to guide in the design of peptidic inhibitors of suitable size, length, and specific sequence for binding with target proteins. Without any prior structural knowledge or a complex structure to guide us, we have to use a *de novo* approach by beginning with the random generation of all possible peptide candidates starting from dipeptide, tripeptide, tetrapeptide and so on. The HIPe contains 28 amino acid residues thus there are at least 20^28^ peptide candidates. To *in silico* create and/or experimentally synthesize these enormous amounts of peptides and then investigate their binding affinity either by computational or experimental methods with histone H4 is clearly impossible. By utilisation of the histone H4-histone acetyltransferase complex, as we demonstrate in this work, only 9 peptides were *in silico* designed, which were extracted from more than a trillion candidates, and 4 peptides were synthesised and next experimentally tested. Also, by use of a structure-based method we were able to identify the peptide with a specific sequence (HIPe) that can bind histone H4 and exhibited biological activity in various experiments. On the other hand, a random peptide, such as a scrambled version of HIPe (sHIPe) which has the same length, same amino acid composition, and therefore, importantly, the same overall net charge as HIPe, cannot bind and neutralise histone H4 in an *in vivo* model as shown in our previous study [17]. Among a trillion candidates in the chemical space of peptides, there might be also other potent inhibitors, but to find the most potent one is absolutely challenging. However, a rational approach by utilisation of structure-based methods, as presented here and also in our previous work [28], [33], assists us to rapidly identify lead compounds/peptides which can also be exploited by further structure–activity relationship study for structural optimisation to improve binding affinity and specificity.

To verify the application of structure-based methods for the development of inhibitors for IDPs/IDPRs, we also utilised similar approaches to design and develop inhibitors for histone H2A (Fig. 6). A complex between bromodomain and histone H2A was used as a starting structure. By use of various computational methods in different steps as explained above, we have *in silico* designed in total 12 peptides (Table 3). Again, we used a fast and inexpensive MM/GBSA method to predict binding affinity of these peptides, and we selected only 5 peptides (cyclic-6, −7, −8, −9 and −17) for further synthesis and functional characterisation. The most active peptide (cyclic-17 or called CHIP) exhibited inhibitory activity in both *in vitro* and *in vivo* experiments; more details of the experimental results can be found in our recent publication [18]. Identification of CHIP to neutralise histone H2A proves and strengthens the usefulness and robustness of the structure-based method to developed (specific) inhibitors for disordered proteins. The N-terminal domain of histone H2A (^1^SGRGKQGGKARAKAKTRSSR^20^) contains several positively charged (arginine (R) and lysine (K)) and partially charged (serine (S) and threonine (T)) residues; theoretically, peptides with high overall negative net charge (cyclic-8 > cyclic-7 ~ cyclic-9 > cyclic-6 > cyclic-17) should exhibit a strong binding with histone H2A. However, experimental results (section 2.6 and Fig. 7) revealed that only cyclic-17 (CHIP) can statistically significantly reduce monocyte adhesion; thus, CHIP represents a strong binder with histone H2A. As demonstrated here, by utilisation of the bromodomain-H2A peptide complex (PDB ID: 4QYL), we have successfully developed the potent peptidic inhibitor (CHIP) for binding with histone H2A. The strategy applied here is to combine simple and fast, yet reliable methods: computational approaches such as molecular docking and short conventional MD simulations that generate a structural framework/context with inexpensive binding free energy calculations (MM/GBSA) method) that are able to prioritize the most interesting structures by distinguishing between lower and higher affinity conformations. Most importantly, the computational approaches as presented in this work can significantly reduce time and cost required in drug discovery and development campaigns, and we believe that our protocols can be widely applied to develop novel inhibitors for other IDPs/IDPRs as well. However, one has to keep in mind that we present here a “general workflow” (not a “gold standard” protocol) to develop peptidic inhibitors for disordered proteins. Specific parameters and protocols (i.e., force fields, simulation times, binding free energy methods as well as docking & simulation programs) can be adjusted according to the investigated systems, to available structure–function information and also to what is convenient (availability of programs) for the users.

During the last decade, histones have increasingly become a research topic in life sciences. Not only their roles in post-translational modification and chromatin packaging or their implication in epigenetics but the discovery of their cytotoxicity has sparked considerable interests from different research fields. Histones, especially the core histones (H2A, H2B, H3, and H4) that are released from or reside in NETs are challenging drug targets. These core histones share similar secondary structures (alpha helices at the core domain and unstructured tail at the N-terminus with several positively charged residues) but their cytotoxicity effects to specific cell types and tissues remain elusive. When present in the nucleosome they exhibit moderate cytotoxicity; however, free histones can be lethal to an organism within minutes and each subtype shows specific cytotoxicity to different cell types and tissues [42], [43]. For instance, we and other research groups have discovered that plasma levels of extracellular histone H3 correlate with mortality in sepsis patients [44], [45]. On the other hand, as reported in our recent publication, histone H4 is the major mediator causing cytotoxicity in cardiac smooth muscle cells (SMCs) [17]. Also, it was reported that only histone H4, but not other subtypes, plays a key role in platelet aggregation in thrombocytopenia [46]. Thus, specific inhibitors for each histone subtype are needed. The histone H4 as well as H2A inhibitor developed from our work can be additionally utilised as starting structures for further development of specific inhibitors for each histone subtype by employing structure-based methods as demonstrated here.

Furthermore, several recent studies have revealed a role for NETs in the pathogenesis of COVID-19 [47], [48], [49], [50]. Thus, NETs may represent a novel and alternative target for COVID-19, and the histone inhibitors developed from our work might likely be used to neutralise histone H2A or H4 residing in NETs and consequently they may be applied to prevent the propagation of COVID-19. Infection by the severe acute respiratory syndrome coronavirus 2 (SARS-CoV-2), the virus that causes COVID-19, can progress into acute respiratory distress syndrome (ARDS) which is a severe condition that can lead to death [51]. ARDS is also commonly found in other viral diseases such as in MERS and SARS, and ARDS represents the leading cause of mortality in these diseases caused by the infection of coronaviruses [51], [52]. Development of inhibitors targeting proteins that play a crucial role in the pathogenesis of ARDS represents an alternative approach to combat COVID-19. Several studies have revealed that extracellular histones are key players in the pathogenesis of ARDS and are promising biomarkers and drug targets for ARDS [53], [54], [55]. Thus, these histone inhibitors (HIPe and CHIP) can additionally be exploited as a structural basis for further development into drugs or molecular diagnostic tools for COVID-19-associated ARDS.

## 4 Conclusion

In this work, we have demonstrated our proof of concepts; *i.*) disordered proteins (e.g., histone H4 and H2A) represent druggable targets and *ii.*) structure-based methods can be utilised to develop inhibitors for IDPs/IDPRs. The successes of development of novel peptidic histone H4 (HIPe) and H2A (CHIP) inhibitors will pave a way for us and for other drug researchers to expand the plethora of drug targets to other histone subtypes as well as to other disordered proteins. We have successfully applied computational methods to develop HIPe which exhibits high potential to inhibit histone H4-induced cell lysis and consequently shows therapeutic benefit effects in an atherosclerotic mouse model. By using similar computational methods, we have developed CHIP, a therapeutic peptide capable of targeting NET-resident histone H2A, and CHIP shows a potential to reduce monocyte adhesion. The protocols and computational approaches used here are fast, simple and straightforward methods which can also be applied to identify and develop novel inhibitors for other IDPs/IDPRs drug targets. Taken together, we not only provide a proof of concept by development of inhibitors to target disordered proteins, but we also provide novel technologies and simple methods to develop inhibitors for IDPs/IDPRs. Furthermore, HIPe and CHIP can be exploited for further development into drugs to treat histone- and/or NETs-mediated diseases such as sepsis, atherosclerosis, acute lung injury, ARDS, and inflammatory diseases.

## 5 Materials and methods

### 5.1 Molecular dynamics (MD) simulations of histone H4-Membranes and histone H4-peptide-Membranes complexes

The coordinates and 3D structure of histone H4 were obtained from the PDB [56] (Chain F of PDB ID: 1KX5). To investigate the binding mechanism between histone H4 and membrane, we first constructed the H4-membrane complex by use of the membrane builder module available in the CHARMM-GUI website [57]. The upper layer of membrane contains 251 units of DOPC lipids whereas lower layer composes of a mixture of DOPS (50 units) and DOPC (200 units) lipids. Histone H4 was put above the upper membrane (approximately 20 Å from the membrane surface), and to solvate the complex waters were then added by setting water thickness to 35 Å from the membrane layers and NaCl was also added to set the final concentration of the system to 0.15 M. The same set up was also applied for the complex between Histone H4-HIPe (peptidic inhibitor) and membrane.

These derived complexes were then subjected to molecular dynamics (MD) simulations by applying CHARMM36 force field (C36 FF) derived from the CHARMM-GUI website and using AMBER16 program to perform MD simulations. Prior to running MD simulations, the systems were first relaxed by employing an energy minimisation (2,500 steps of steepest descent followed by 2,500 steps of conjugate gradient algorithm). During this phase, histone H4 (and the HIPe peptide) were constrained by application of a soft position constraint (10 kcal/mol·Å^2^) while membranes were partially fixed using a force constraint of 2.5 kcal/mol·Å^2^. After the completion of energy minimisation, a position-restrained MD phase was employed for 400 ps. During this period, consecutive reduced values of force constraint were also applied to slowly relax the complexes; starting from 10, to 5, 2.5, 0.5, and 0.1 kcal/mol·Å^2^ for protein and peptide while applying a force constraint of 2.5 to 1, 0.5, and 0.1 kcal/mol·Å^2^ for the membrane. The temperature of the complexes was set to 303.15 K by continuously increasing the temperature from 0 to this value and was then kept fixed at 303.15 K by using Langevin dynamics with a collision frequency of 1 ps^−1^. Finally, free MD simulation (without any position or force constraints) was carried out for 50 ns and in order to efficiently explore the binding event in a short time scale of MD simulation, the sampling power of accelerated MD (aMD) [58] simulation was subsequently employed from 50 to 200 ns. The MD simulation of histone H4-HIPe-membrane complex was extended to 250 ns and the aMD approach was also applied from 50 to 250 ns. The same parameters were used for free MD and aMD simulations by setting temperature at 303.15 K, pressure at 1 bar, and time step of 2 fs with SHAKE algorithm to satisfy the bond distance and geometry during the MD simulation. The particle-mesh Ewald (PME) method was used to compute electrostatic interactions whereas the cut-off setting at 12 Å with the force-based switching at 10 Å was used to calculate non-bonded interactions.

### 5.2 Structure-based approaches for rational peptidic inhibitor design

The generic structure-based approach utilised to design and develop inhibitors targeting the N-terminal domain of histone H4 is illustrated in Fig. 8. Also, the schematic representation of the workflow to generate peptidic inhibitors for histone H2A is summarised in Fig. 6.

#### 5.2.1 Identification of complexes containing histone H4 or H2A

First, we searched for a complex between N-terminal domain of histone H4 in complex with its binding protein partners from the PDB and the complex between histone H4 (*Ophiophagus hannah*) and histone acetyltransferase type B (PDB ID: 4PSW) was selected (Step 1, Fig. 8). The X-ray structure of Homo sapiens (human) histone H4 was extracted from nucleosome structure (chain B of PDB ID: 1KX5) and was subjected to MD simulations (Step 2, Fig. 8) to investigate secondary structure of histone H4 and generate diverse conformations of histone H4. Similar protocols as in our previous work [33] were applied for MD simulations. Briefly, AMBER14SB force field implemented AMBER16 program were assigned for the proteins and standard parameters (i.e., temperature (300 K), pressure (1 bar), time step of MD simulation (2 fs), and SHAKE constraint) and TIP3P water model were used. MD simulations of free histone H4 were carried out for 100 ns and snapshots were extracted and inspected for its secondary structure. The structures of the N-terminal tail of histone H4 showing linear conformation (likely to fit into the binding pocket of acetyltransferase) were selected. Then, binding modes of human histone H4 with acetyltransferase were determined by application of protein–protein docking using the HADDOCK2.2 webserver [59] (Step 3, Fig. 8).

Similar approaches were applied to generate a complex between histone H2A and its binding partner. First, MD simulation of free histone H2A (chain C of PDB ID: 1KX5) was employed for 100 ns by using the same force fields and parameters as described above. Then, the structures of histone H2A displaying linear conformation at the N-terminus were selected and consequently were superimposed on H2A-derived peptide fragment which is in complex with bromodomain (PDB ID: 4QYL).

#### 5.2.2 Binding free energy (BFE) calculation and peptide design

The derived docking poses between histone H4 and acetyltransferase obtained from protein-protein docking in the previous step (in total 33 different binding modes) were refined by energy minimisation and short MD simulation (500 ps) using same parameters and protocols as described above. Subsequently, binding free energy (BFE) of these complexes were computed by extracting snapshots from 0 to 500 ps and using MMPBSA.py module implemented in AMBER16 program. Default parameters of molecular mechanics/generalised Born surface area (MM/GBSA) of GB model 8 were used for BFE calculation. Since we intended to distinguish likely from less likely poses and/or peptide ligands, the relative BFE was considered, thus BFE values were approximated from the enthalpy value and the entropy term can be ignored. More details of BFE and MM/GBSA method can be found in our previous publication [33]. The docking pose that gave the lowest BFE values, indicating a thermodynamically favourable pose, was selected as a starting structure for further rational peptide design (Step 4, Fig. 8). MD simulation of the selected complex was extended for 20 ns to investigate the stability of complex as well as to identify key residues and interactions. The chosen binding mode showed that the peptide fragment (residue 162 to 173) of histone acetyltransferase is the major part that binds and interacts with the disordered N-terminal histone H4. Thus, this sequence (EAANYIDETDPS) was extracted and served as a starting structure for peptide design. In total, we designed 9 different cyclic peptides. The binding mode of peptides and histone H4 were predicted by using the HADDOCK2.2 webserver [59]. Then, MD simulations of the docking poses of histone H4-peptide complexes were employed for 500 ps and finally BFE of these peptides was computed (Step 5, Fig. 8). The same parameters and protocols (MD inputs, force fields, parameters and protocols and MM/GBSA method using GB model 8) as in the previous step were applied.

A complex between histone H2A and the human bromodomain were subjected to structural optimisation by performing an energy minimisation and MD simulations (20 ns), using the same parameters and protocols as explained above. DC per residue and structural analysis pointed out that residue 31–43 and 78–91 of the bromodomain are the key residues to contribute to interaction with histone H2A, thus these residues were selected as a template to design peptidic inhibitors for histone H2A. Again, rational approaches were utilised to design peptides to improve their binding and interactions with histone H2A, and binding mode and binding free energy of the designed peptides with histone H2A were predicted by use of HADDOCK2.2 webserver and MM/GBSA method (GB model 8) as described above.

For both cases (design of peptides for histone H4 and H2A), the peptides that showed lowest BFE (more negative values) were selected for synthesis and further biological activity testing in different experiments in the next steps. Peptides used in this study were purchased from a commercial vendor (Pepscan Therapeutics, Lelystad, The Netherlands) and were synthesised by standard Fmoc solid phase peptide chemistry to a purity > 90%.

### 5.3 Histone H4 killing assays

More details of experimental methods (cell culture and activation and cell viability assay) can be found in our recent publication [17]. Briefly, mouse vascular aorta/smooth muscle cells (MOVAS) were cultured overnight in a 96-well plate in a complete medium: DMEM + G418 (0,2 mg/mL), 1 mM sodium Pyruvate, 10% FCS at the density of 5000–10000 MOVAS per well. After 24 h of incubation, the MOVAS were washed and stimulated by 50 ug/mL recombinant histone H4 in medium without FCS. To test the potential of candidate peptides for reducing cytotoxicity induced by histone H4, the peptides (cyclic-6, cyclic-7, cyclic-8, and cyclic-9) were first incubated with histone H4 for 1 h at room temperature before adding to the cells. Different concentration of peptide (1, 10 or 100 µg/mL) were tested. Instead of histone H4 or histone H4 and peptides, phosphate buffer was added to medium to use as a control experiment. After 1 h of incubation, medium was removed and cells were stained for 5–10 min with PI (propidium iodide) (1 mM) in 50 μL medium (without phenol red), after that another 50 μL medium (without phenol red) containing 10 mM Sytogreen was added. Both incubations were performed at 37 degrees. Then, 5 min later cell death was finally assessed by inverted fluorescence microscopy.

### 5.4 Blocking monocyte adhesion by designed peptides (*In vitro* adhesion assay)

More details of the *in vitro* adhesion assay are comprehensively explained in our recent publication [18]. Shortly, we investigated the adhesion of human classical monocyes to NETs under static conditions. To this end, we added CellTrace calcein violet labelled monocytes (0.5x10^5^ cell/well) to flat bottom 96-well plates with neutrophils (2x10^5^ cells/well) adherent at the bottom. Where indicated NETs were induced by addition of 25 µM calcium ionophore A23187. After 15 min of adhesion, non-adherent monocytes were washed off and adherent monocytes were quantified by using a microplate reader (Tecan infinite™ 200 pro). To investigate an activity of the designed peptides to prevent monocyte adhesion to NETs, we first incubated NETs and the peptides (cyclic-6, −7, −8, −9 and −17 at the concentration of 200 μg/ml) together and then added monocytes by following the described protocols [18].

## 6 Disclosures

K.W., O.S. and G.N. are inventors of a patent application owned by the Maastricht University on targeting histones in cardiovascular inflammation.

## Author contributions

K. W. planned, designed and performed the computational parts and wrote the manuscript. C. S.-R., Q. B., and A. S. performed the experiments, analysed and interpreted experimental data. O. S. and G. A. F. N. supervised specific data acquisition and analysis, proof-read the manuscript and provided funding.

## Declaration of Competing Interest

The authors declare that they have no known competing financial interests or personal relationships that could have appeared to influence the work reported in this paper.
