# Supplementary material for: Structure-based peptide design targeting intrinsically disordered proteins: Novel histone H4 and H2A peptidic inhibitors
Source: Comput Struct Biotechnol J. 2021 Jan 21;19:934–48. doi: 10.1016/j.csbj.2021.01.026 (PMC7856395; doi:10.1016/j.csbj.2021.01.026)
Supplement: Supplementary data 1 [file mmc1.docx]

**Supplementary Information**

**Structure-Based Peptide Design Targeting Intrinsically Disordered Proteins: Novel Histone H4 and H2A Peptidic Inhibitors**

Kanin Wichapong^1,*^, Carlos Silvestre-Roig^2,3^, Quinte Braster^2,3^, Ariane Schumski^2,3^, Oliver Soehnlein^2,3,4^, Gerry A. F. Nicolaes^1^

^1^Department of Biochemistry, Cardiovascular Research Institute Maastricht (CARIM), Maastricht University, Maastricht, The Netherlands

^2^Institute for Cardiovascular Prevention (IPEK), LMU Munich Hospital, Munich, Germany

^3^German Center for Cardiovascular Research (DZHK), Partner Site Munich Heart Alliance (MHA), Munich, Germany

^4^Department of Physiology and Pharmacology (FyFa), Karolinska Institute, Stockholm, Sweden

***Corresponding author**

**Kanin Wichapong, Ph.D.**

Department of Biochemistry, Cardiovascular Research Institute Maastricht (CARIM), Maastricht University, The Netherlands

**Phone:** +31-43-3884363; **Fax**: +31-43-3884159

**E-mail**: k.wichapong@maastrichtuniversity.nl, kanin.wichapong@gmail.com

**Table SI-1.** Binding Free Energy (kcal/mol) of different binding mode between human histone H4 and histone acetyltransferase

| Complex Number | Binding Free Energy (kcal/mol) |
| --- | --- |
| 1 | -49.96 ± 4.98 |
| 2 | -76.85 ± 5.89 |
| 3 | -55.30 ± 8.00 |
| 4 | -62.30 ± 3.72 |
| 5 | -55.30 ± 7.25 |
| 6 | -68.53 ± 8.17 |
| 7 | -49.30 ± 4.59 |
| 8 | -73.55 ± 6.46 |
| 9 | -30.19 ± 5.59 |
| 10 | -50.23 ± 5.53 |
| 11 | -62.74 ± 5.52 |
| 12 | -40.59 ± 5.05 |
| 13 | -76.61 ± 6.74 |
| 14 | -37.99 ± 6.90 |
| 15 | -54.38 ± 8.44 |
| 16 | -51.71 ± 11.19 |
| 17 | -43.24 ± 6.66 |
| **18** | **-79.84 ± 3.96** |
| 19 | -72.98 ± 4.46 |
| 20 | -63.88 ± 5.34 |
| 21 | -46.55 ± 7.59 |
| 22 | -44.72 ± 4.94 |
| 23 | -34.95 ± 6.11 |
| 24 | -64.61 ± 4.64 |
| 25 | -57.95 ± 8.67 |
| 26 | -59.86 ± 3.51 |
| 27 | -26.06 ± 4.07 |
| 28 | -69.08 ± 4.79 |
| 29 | -58.21 ± 6.05 |
| 30 | -64.41 ± 7.39 |
| 31 | -69.02 ± 3.43 |
| 32 | -41.88 ± 4.06 |
| 33 | -71.45 ± 6.87 |


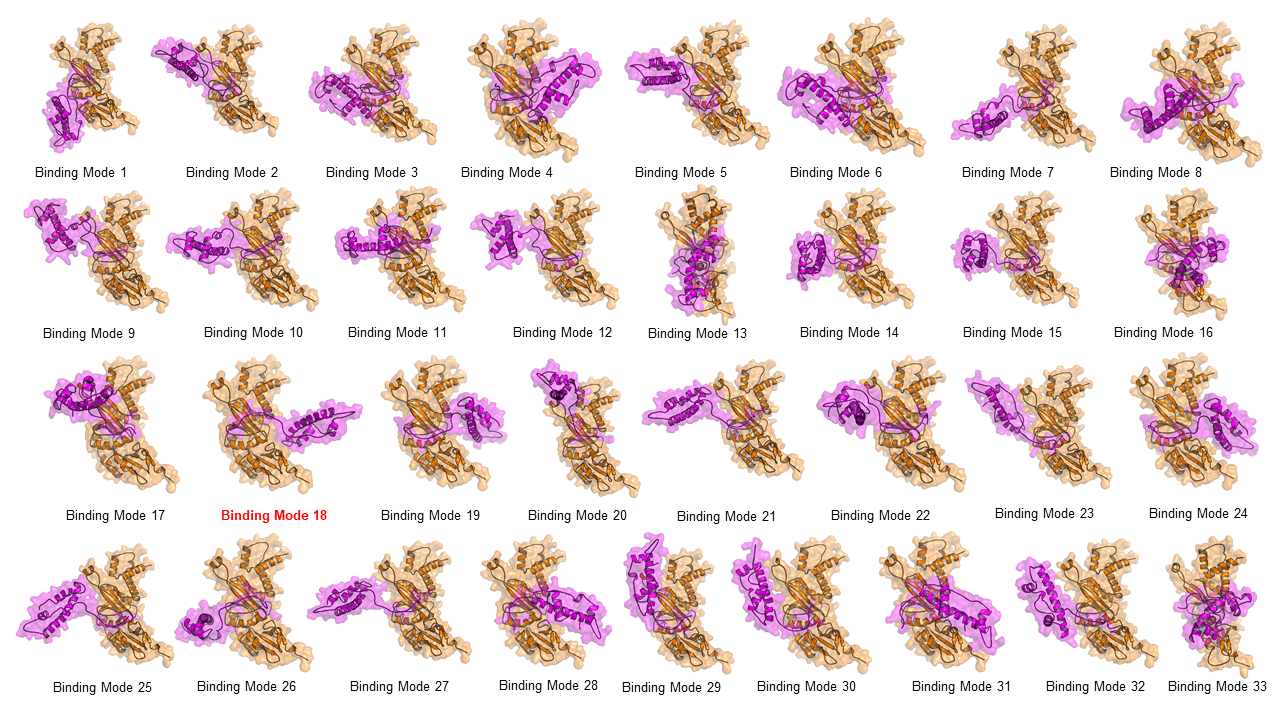


**Figure SI-1.** different binding mode between human histone H4 and histone acetyltransferase.
